# Adding a New Dimension to PH Imaging

**DOI:** 10.1002/pul2.70044

**Published:** 2025-01-23

**Authors:** Andrew Bradley, Mardi Gomberg‐Maitland

**Affiliations:** ^1^ Department of Medicine, Cardiology George Washington University School of Medicine and Health Sciences Washington District of Columbia USA

Increased pulmonary artery systolic pressure (PASP) with downstream consequences on the right ventricle (RV) is the shared hallmark of the various forms of pulmonary hypertension (PH). The underlying pathophysiology of elevated PASP varies greatly between PH classification groups. Right heart catheterization (RHC) is crucial for accurate diagnosis and has significant prognostic and therapeutic implications for patients. In this week's journal, Kim et al. propose a less invasive approach by suggesting the utilization of 4D flow MRI (4D MRI) as a diagnostic tool to differentiate between pulmonary arterial hypertension (PAH) and pulmonary hypertension‐heart failure preserved ejection fraction (PH‐HFpEF) [1].

4D MRI enables the flow of blood within the cardiovascular circuit to be visualized and analyzed in the three physical axes with respect to time. It has been used to characterize abnormal flow patterns in conditions such as aortic stenosis and post transthoracic endovascular aortic repair [2, 3]. Beyond colorful pictures, 4D MRI also provides quantitative measurements relating to the blood's flow including wall shear stress (WSS), peak velocity (*V*_peak_), vorticity (a measure of blood flow rotation), and pressure gradients between cardiac chambers [4]. Earlier work in 4D MRI described scan acquisitions lasting well over 30 min [5]. Ongoing research in MRI sequence development has demonstrated the feasibility of acquiring these 4D data sets in under 15 min, important for patient comfort and clinical throughput [4, 6].

The authors found a decrease in 4D MRI‐derived WSS and *V*_peak_ in patients with PAH versus PH‐HFpEF. Importantly, the patients had similar cardiac output at RHC and metrics of right ventricular contractility on echocardiography. From this standpoint, the study suggests that 4D MRI can identify differences between these two very different pathologies. For a patient hesitant to undergo invasive testing via RHC, data from 4D MRI might yield useful material for a clinic discussion and shared decision‐making.

Not surprisingly, the two groups differed greatly in PASP and pulmonary vascular resistance limiting their results. Despite similar cardiac outputs at RHC, the PAH group had a non‐statistically significant larger PA cross‐sectional area, affecting velocity profiles through the PA. They also had a non‐statistically significant difference toward smaller left ventricular size suggesting chronic RV remodeling. This raises questions about the significance of differences in WSS and *V*_peak_. Are these intrinsic differences in PAH or reflective of remodeled PA flow in response to elevated PASP? Answering this question will be challenging. Future work could involve 4D flow analysis in PASP‐matched PH patients or prospectively following a PH cohort with 4D flow MRI during treatment to identify viable clinical trial MRI‐based endpoints. Larger trials demonstrating clear utility would also help inform payors’ coverage policies.

Despite limitations of between‐group differences and the inability to separate cause from effect, this study offers valuable insights for the PH community. It identifies 4D flow MRI as a feasible method to detect between‐group differences in PH patients. Hypothesis‐generating, it suggests different PH triggers affect flow profiles in the PA. 4D MRI flow has potential diagnostic applications and justifies is a potential endpoint for tracking patient response to therapy in PH.

## Author Contributions

Both contributed to all aspects of the manuscript.

## Ethics Statement

The authors have nothing to report.

## Conflicts of Interest

The authors declare no conflicts of interest.

## Data Availability

The authors have nothing to report.
